# Rationale and design of the SPRING trail: effectivity and safety of Pfo closuRe vs medIcine in alleviatiNg migraine, a multicenter, randomized and open-label trail

**DOI:** 10.1186/s12872-024-03866-3

**Published:** 2024-04-05

**Authors:** Yang Zi-yang, Li Hezhi, Luo Dongling, Wang Ximeng, Zhang Caojin, for the SPRING Investigators, Chen Weibin, Guo Tao, Cui Tongtao, Wang Zhanhang, Xiong Zhaojun, Gao Hanhua, Lai Junxing, Yuan Jie, Chen Jianying, Wang Xiaodong, Liu Wei, Zhang Hongwei, Zhang Gangcheng, Zheng Xuan, Shen Qunshan, Chen Xiaobin, Xie Dujiang, Zhang Wenqi, Wang Zhongchao, Wei Wenbin, Zhou Yang, Zhang Wei

**Affiliations:** 1 Guangdong Cardiovascular Institute, Guangdong Provincial People’s Hospital (Guangdong Academy of Medical Sciences), Southern Medical University, Zhong Shan Er Road No. 106, Yue Xiu District, Guangzhou, Guangdong 510080 China; 2 Guangdong Provincial Key Laboratory of South China Structural Heart Disease, Guangzhou, Guangdong China; 3 Global Health Research Center, Guangdong Cardiovascular Institute, Guangdong Provincial People’s Hospital (Guangdong Academy of Medical Sciences), Southern Medical University, Guangzhou, Guangdong China

**Keywords:** Randomized clinical trial, Migraine, Patent foramen ovale

## Abstract

**Background:**

Migraine is a leading cause of disability worldwide. Several retrospective studies have suggested that the closure of the Patent Foramen Ovale (PFO) may provide relief from migraines. However, three randomized controlled trials did not meet their primary endpoints regarding migraine cessation, reduction in monthly migraine days, and responder rates.

**Methods:**

The SPRING study is a multicenter, prospective, randomized, and open-label trial designed to compare the effectiveness and safety of PFO closure versus medication in the relief of migraines. The primary endpoint is the total cessation of migraines, as recorded in patient headache diaries during the follow-up period. Additional diagnostic tools include echocardiography with agitated saline contrast, transcranial Doppler, and routine laboratory measurements.

**Conclusion:**

The SPRING trial aims to assess the effectiveness and safety of PFO closure versus medication in mitigating migraines in real-world settings. (Clinical Trails ID: NCT04946734).

## Background

Migraine is recognized the leading cause of disability among individuals under 50, as reported by the World Health Organization's Global Burden of Diseases Study, 2019 [1]. Despite its prevalence, advancements in migraine management have remained relatively stagnant since the 1990s [1]. While effective symptomatic and preventive treatments exist, none can substantially attenuate migraine symptoms. Over the past two decades, numerous studies have indicated an association between migraine risk and the presence of Patent Foramen Ovale (PFO) and its right-left shunt grade, suggesting PFO as a potential causative factor [2–5]. Proposed mechanisms for this correlation include hypoxia, changes in cerebral autonomic regulation, and venous thromboembolism due to right-left shunting and in situ thrombosis within the PFO [6–9]. Furthermore, multiple studies have demonstrated that device closure of PFO may result in migraine relief [10–13]. However, none of the three randomized controlled trials (RCTs) on PFO closure achieved their primary endpoints, including migraine cessation, reduction in monthly migraine days, and responder rates [14–16]. Consequently, current guidelines provide minimal endorsement for PFO closure as a treatment for migraine due to inconsistencies between RCTs and retrospective studies. Recognizing the need for a comprehensive evaluation of PFO closure's efficacy in preventing migraines, we have launched a real-world design RCT. This trial aims to compare the effectiveness and safety of PFO closure versus medication in alleviating migraines.

### Study design

The Spring study is a multicenter, randomized and open-label trail comparing the effectivity and safety of PFO closure vs medicine in alleviating migraine, with a follow-up period of 12 months (ClinicalTrials.gov ID NCT04946734). The trial is performed in 21 centers in China and has been approved by all local ethics committees. Our hypothesis posits that, compared to medication alone (clopidogrel 75 mg q.d. and aspirin 100 mg q.d.), PFO closure accompanied by one months of clopidogrel (75 mg q.d.) and six months of Aspirin (100 mg q.d.) will be more effective in completely ceasing migraines. Patient recruitment is expected to be completed by the end of 2024, with final results anticipated in 2025.

Patients with migraine and PFO were included in this study. Participant inclusion and exclusion criteria are presented in Table 1.

**Table 1:** Inclusion and exclusion criteria

Inclusion criteria
1. Subject is aged between 16 and 60 years old.
2. Subject is diagnosed of PFO with right-to-left shunt, confirmed by Transthoracic echocardiography (TTE) or transesophageal echocardiography (TEE) with agitated saline contrast.
3. Subject is diagnosed of right-to-left shunt, confirmed by transcranial doppler with agitated saline contrast.
4. Subject is diagnosed of migraine without identifiable cause of headache.
5. Subject signs an informed Consent Form and is willing to participate in follow-up visits; 6. Primary and secondary endpoints.
Exclusion criteria:
1. Subject is diagnosed of headache with clear etiology.
2. Subject had cerebral hemorrhage, bleeding events in other organs within 3 months or was in high risk of bleeding.
3. Subject presents with ischemic lesions as indicated by brain CT/MR imaging.
4. Subject is diagnosed of hepatic insufficiency: ALT or AST > 3 × ULN at the screening visit.
5. Subject is diagnosed of moderate to severe renal insufficiency: eGFR < 30 ml/min/1.73m2 at the screening visit.
6. Subject has uncontrolled arrhythmia with clinical significance within 90 days.
7. Subject is diagnosed of unstable angina, severe coronary atherosclerosis or myocardial infarction within 90 days.
8. Subject is diagnosed of pulmonary artery embolism, peripheral artery embolism or deep Vein Thrombosis.
9. Subject cannot follow the study procedure due to other acute or chronic diseases.
10. Subject is pregnant or lactating.
11. Subject is under other RCT.
12. Subject has a life expectancy < 1 year.
13. Subject cannot follow the study procedure due to other reasons in the opinion of the investigators.

### Study protocol

**Fig. 1:**
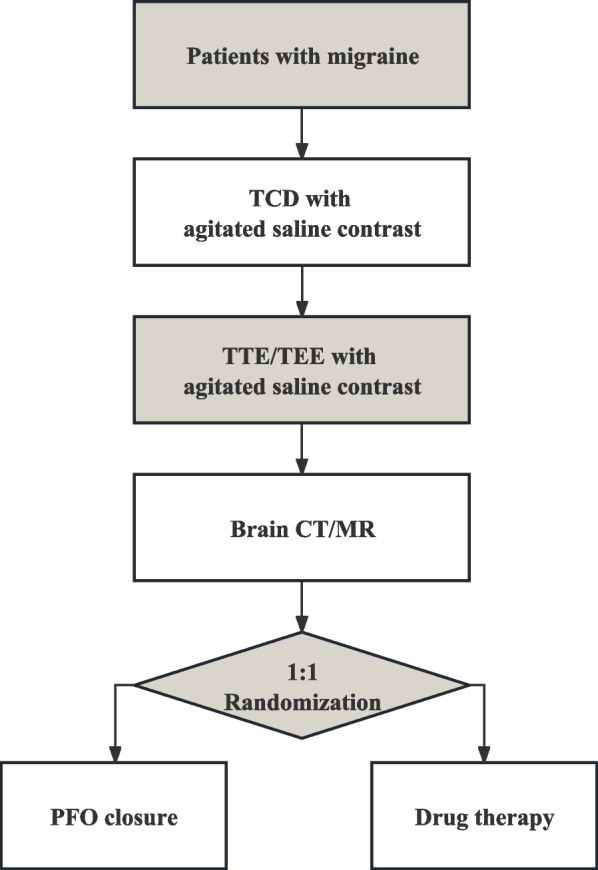
Flow chart of the study design. This figure explains the flow chart of the inclusion criteria screening and randomization

The flow chart of the study design is presented in Fig. 1. Once patients are diagnosed with migraine and PFO, a right-to-left shunt is evaluated using transcranial Doppler (TCD) and transthoracic/transesophageal echocardiography (TTE/TEE) with agitated saline contrast. Upon confirmation of the shunt and PFO, investigators will detail the study protocol to the patients. Following their agreement and the signing of the consent form, we will collect routine laboratory test results, brain imaging (CT/MR), rating scale questionnaires, and other baseline characteristics.

After a comprehensive review of inclusion and exclusion criteria, the investigators will proceed with the randomization and distribute headache diaries. Patients in the control group will be prescribed aspirin (100 mg daily) for 6 months and clopidogrel (75 mg daily) for 1 month. In contrast, patients in the treatment group will undergo percutaneous PFO closure in the catheterization laboratory.

### PFO device closure

After local anesthesia, a 6-French MPA 2 catheter was inserted into the right heart system through the right femoral vein by placement of a 6-French vascular sheath. Under fluoroscopy, we measured the pressure in right atrium and pulmonary artery to exclude pulmonary hypertension related PFO. Then, we advanced the catheter through the PFO and anchor in the left inferior pulmonary vein. After accessing the size and location of the PFO, the MPA 2 catheter was exchanged for an 8-French or 9-French delivery sheath and a PFO closure device would be placed across the atrium septum under bedside TTE and fluoroscopy. After successful implement, patients were prescribed same medication of Aspirin 100 mg qd for 6 months and clopidogrel 75 mg qd for 1 month, and would be usually discharged home in the next day.

The success of the percutaneous PFO closure procedure will be defined as implantation of the PFO device without any major complication including death, stroke, or any complication that requires surgical or endovascular treatment.

### Follow up

Follow-up assessments will be conducted at 1, 3, 6, and 12 months post-intervention. These will include the recording of migraine diaries, completion of questionnaires assessing migraine impact, quality of life, medication history, and any serious adverse events. At the 6 and 12-month marks, or upon reaching the primary endpoint, additional follow-up assessments will be conducted. These will consist of TTE/TEE with right heart contrast and TCD with a bubble test to re-evaluate the right-to-left shunt. When adverse events are identified during follow-up, investigators will assess the condition and severity to determine whether to terminate the trial.

### Study outcomes

Primary and secondary endpoints are presents in Table 2. The outcomes of the migraine changes are adjudicated based on the headache diary data. The total days of migraine is defined as migraine frequency per month plus migraine average duration, divided by 24, round up to nearest integral. The definition of responder rate is the percentage of patients who have at least 50% reduction of monthly migraine attacks from their baseline. Other efficacy measures include migraine severity, as assessed by the HIT-6 scale [17], and quality of life alterations, determined by the SF-36v2 questionnaire [18]. These changes would be also analyzed the relationship with a successful PFO closure and residual shunt.

**Table 2:** Primary and secondary endpoints

Primary endpoint
Complete cessation of migraine during the follow up.
Secondary efficacy endpoints
Mean reduction of monthly migraine attacks. Mean reduction of monthly migraine days. Responder rate.
Secondary safety endpoints
All-cause mortality. Peripheral embolism. New-onset arrhythmia warranting intervention. New-onset myocardial infarction. Other complications related to PFO closure procedure, device and antiplatelet drugs. Other side effects associated with the drugs used in the study.

### Data management and monitoring

All clinical data will be de-identified and stored in an electronic data capture system. The Headache Diary Review Committee, blinded to the randomization, will oversee the calculation and recording of migraine severity and frequency. The Independent Event Committee and the Data Safety Monitoring Board will ensure the integrity of the data and monitor the safety of the participants.

### Sample size and statistical considerations

This was a randomized controlled study with PFO blocking group as the intervention group and drug treatment group as the control group. Migraine frequency (monthly migraine days), improvement in migraine frequency, severity (migraine scale score) and quality of life score were the outcome indicators. According to previous reports, the frequency of migraines treated with medication was 6.5 ± 2.4 days/month. The migraine HIT-6 score was 66.2 ± 5.1; After treatment, the number of migraine attacks per month can be reduced by 25%; SF12 quality of life score increased by 1.2 ± 6.9 points after treatment. The frequency of migraine was expected to decrease by 0.6 days in PFO occlusion group. Migraine scale HIT-6 score decreased by 1.5 points; After treatment, the number of migraine attacks per month can be reduced by 50%; SF12 quality of life score improved by 4.2 points after treatment. The test level was set as bilateral α = 0.05, and the assurance was 80%. PASS 15.0 software was used to calculate the required sample size of 113, 183, 60 and 156 cases in each group based on the above four observation indicators. Taking the maximum sample size and considering 20% of the cases of loss and refusal to visit, the final requirement was 220 cases in the PFO blocking group and 220 cases in the drug treatment group.

The primary endpoints will be analyzed across different analysis sets: Intention-to-Treat, per-protocol, and as-treated, with the primary analysis set being Intention-to-Treat. Categorical variables will be described using rates and composition ratios. Group differences in variables such as migraine termination, sociodemographic factors, migraine incidence rate, changes in migraine characteristics (with or without aura and their changes), effective closure rates, or residual leakage will be compared using the chi-square test. Continuous variables will be characterized based on their data distribution, utilizing means, standard deviations, medians, and interquartile ranges. Group differences in variables such as sociodemographic factors, severity and frequency of migraine attacks, and quality of life scores derived from the SF-36v2 questionnaire will be compared using t-tests or the Wilcoxon test.

Additional analysis will involve covariance analysis to account for factors significantly influencing the observed indicators. Different centers will be treated as random effects, and generalized linear models will be employed to further control for the potential impact of center effects. Stratified analysis will be performed based on factors such as gender and age. Predefined subgroups will include migraine with or without aura. Survival function estimation will be conducted using the Kaplan–Meier method. Relevant covariates will be controlled for using the Cox proportional hazards model, and truncated data will be analyzed accordingly.

For all analyses, a two-tailed *P* value < 0.05 was used as the criterion for statistical significance. Analyses were performed using SPSS 22 software (IBM Corp., Armonk, NY, USA).

## Discussion

This study is a multicenter, randomized trail evaluating the effectivity of PFO closure in alleviating migraine. Previously, the three RCT set their primary endpoints as complete migraine cessation (MIST, 2008) [16], monthly migraine days reduction (PRIMA, 2016) [15] and responder rate (PREMIUM, 2017) [14]. Although some non-randomized studies suggest the efficacy of PFO closure in migraine relief, none of the aforementioned RCTs achieved their set endpoints. In the 2008 MIST study, patients in the closure group were implanted with the STARFlex Technology device, which has been reported with a higher complication. In addition, a relatively shorter follow up time might also underpower the PFO closure effect. While in the PRIMA and PREMIUM study, both use Amplatzer PFO Occluder and had a 12 months follow up time, but still had an in-neglectable frequency of residual shunt (12% & 11% V.S. 6%). Additionally, small sample sizes might have contributed to these studies not meeting their endpoints. Notably, a pooled analysis of PRIMA and PREMIUM demonstrated a significant mean reduction in monthly migraine days, attacks, and an increase in complete cessation rates in the PFO closure groups [19]. A significant discrepancy between RCTs and retrospective studies might stem from the meticulous screening of patients in RCTs for refractoriness to migraine prevention medication, which may not align with real-world clinical practice. To solve these problems, our SPRING study employs a more recent generation of PFO closure devices and strategy, along with a larger sample size. Unlike previous approaches, we are not mandating preventive medication before closure, aiming to assess if patients can achieve migraine relief post-intervention, thus aligning more closely with the scenario when patients first seek medical advice for migraines.

## Conclusion

This multicenter, randomized trial aims to compare the efficacy and safety of PFO closure versus medication in alleviating migraines. Given the inconclusive evidence from previous RCTs, which failed to reach their primary endpoints, our study is designed to provide a robust evaluation of the effectiveness of these treatments in a real-world setting.

## Data Availability

No datasets were generated or analysed during the current study.
